# Placental infections with histologically confirmed *Plasmodium falciparum* are associated with adverse birth outcomes in India: a cross-sectional study

**DOI:** 10.1186/1475-2875-13-232

**Published:** 2014-06-13

**Authors:** Rukhsana Ahmed, Neeru Singh, Feiko O ter Kuile, Praveen K Bharti, Pushpendra P Singh, Meghna Desai, Venkatachalam Udhayakumar, Dianne J Terlouw

**Affiliations:** 1 Department of Clinical Sciences, Liverpool School of Tropical Medicine, Liverpool, UK; 2 National Institute of Malaria Research Field Station, Regional Medical Research Centre, Jabalpur, India; 3 Malaria Branch, Division of Parasitic Diseases and Malaria, Center for Global Health, Centers for Disease Control and Prevention, Atlanta, Georgia, USA; 4 Malawi-Liverpool-Wellcome Trust Clinical Research Programme, Blantyre, Malawi

**Keywords:** Malaria, Pregnancy, Placenta, Histopathology, Birth weight, Haemoglobin, Gestational age, India

## Abstract

**Background:**

Few studies have assessed placental malaria infections from low transmission areas by histopathology to define their impact and underlying mechanisms.

**Methods:**

Peripheral smears and rapid diagnostic tests (RDTs), placental smears and histological samples, birth weight and gestational age were collected from 2,282 deliveries in three hospitals during a one-year (2006–2007) continuous cross-sectional survey in Madhya Pradesh. Placental histopathology included all 50 cases positive by microscopy or RDT plus 456 randomly selected samples of women negative for malaria by microscopy or RDT. Histological examination included parasites, inflammatory cells, pigment in fibrin, and morphological changes.

**Results:**

There were 52 histology-positive cases; 38 (73.1%) active (acute and chronic) and 14 past infections. Intervillous parasitaemia was low (60% had < 1% parasitaemia) and monocytosis mostly mild (63%). Compared with uninfected placentas, acute *Plasmodium falciparum* infections were associated with stillbirth (RR 3.8, 95% CI 1.2-12.1), lower maternal haemoglobin (mean difference: 1.5 g/dL, 95% CI 0.5-2.5), lower birth weight (mean difference 451 g, 95% CI 169–609) and shorter gestation (mean difference 0.8 weeks, 95% CI 0.2-1.4). Chronic or past infections were not associated with these outcomes. Among the 11 peripheral *Plasmodium vivax* cases, placental parasites were absent, but they were associated with increased placental polymorphonuclear cells.

**Conclusions:**

Malaria associated stillbirth and low birth weight in women with low protective immunity may result, at least in part, from a shortened gestation triggered by acute infection, stressing the importance of early malaria detection.

## Background

An estimated 125 million pregnant women worldwide are exposed to the risks of malaria in pregnancy (MiP) every year, contributing to morbidity and mortality in pregnant women and newborn babies [1,2]. The pathology is related to placental sequestration due to cytoadhesion of *P. falciparum* infected erythrocytes to the placental glycosaminoglycan chondroitin sulphate A receptors and associated inflammatory responses. The resulting features of parasite sequestration, increased presence of inflammatory cells, deposition of pigment (haemozoin), and fibrin characterize placental malaria and have been used to classify the chronology of placental infections [3,4]. The association between histologic grades of infection in *P. falciparum* infected placentas and low birth weight and maternal anaemia is well documented in high malaria endemic African regions [5-8], with reports of dense parasitaemia and occasional massive chronic intervillositis and monocyte infiltration mainly, but not exclusively from Africa [9,10]. This led to suggestions that the accumulation of parasites *per se,* with the corresponding inflammatory response to the sequestered parasites is associated with fetal growth restriction [11-13].

Reports from the Asia-Pacific and Latin America question whether the same holds for those regions. In these areas, a detrimental impact has been observed with relatively low-grade and early-pregnancy peripheral *P. falciparum* and *P. vivax* infections and placental malaria described in Colombia [14-18]. Severe manifestations of *P. vivax* infection have been associated with pronounced maternal anaemia and cytokine-mediated inflammatory response [19]. Low-grade *P. vivax* infections have been associated with intrauterine growth restriction [20], and an increased risk of miscarriage and low birth weight [21]. These findings stress the limited understanding of the population burden in areas outside Africa, and have led to recent calls for more area and species specific information to guide control strategies for these highly diverse malaria endemic regions [21].

Comprehensive, reproducible histopathology studies can help elucidate the placental changes and mechanisms that occur with *P. falciparum* and *P. vivax* infections in areas of low transmission. *P. vivax* was not considered to sequester in the microcirculation, until in-vitro studies recently showed that (a fraction of) *P. vivax* infected erythrocytes do have the potential to cytoadhere to the placental syncytiotrophoblast glycosaminoglycans chondroitin sulphate A responsible for *P. falciparum* sequestration in the placenta [22,23]. While this suggests a comparable pathogenesis to *P. falciparum*, the occurrence and extent of sequestration *in vivo* has not been shown. Three other studies recently reported histopathology findings of *Plasmodium* infections in Asia and Latin America [24-26], but overall the number of cases examined remain low (n = 24, 19, and 78 respectively). It is not clear how the morphological changes in the placenta described for placental malaria mainly in Africa [7,9] relate to the commonly used histological grading system for placental malaria [3,4], to other malaria test results and to adverse birth outcome in a low transmission setting [10].

## Methods

### Study location

The study sites were in three districts in Madhya Pradesh, central India. Malaria transmission in this region is highly seasonal and *P. falciparum* and *P. vivax* co-exist. In the underlying burden surveys we conducted the overall maternal malaria prevalence was 1.8% among 2282 women recruited at the time of delivery (0.8% in the dry season and 3.3% in post rainy season). Based on standard microscopy, the prevalence of placental malaria was 1.0%; 0.5% in the dry season and 2.1% in the post rainy season. *P. falciparum* was the predominant species in 72.5% of the observed maternal and 87.0% of placental infections.

According to a previous survey in Madhya Pradesh in 2007–8, 61% of pregnant women visited ANC at least once and 34% at least three times, whereas 47.1% of women had institutional deliveries [27]. The national malaria guidelines at the time of the study recommended parasitological diagnosis with either microscopy or a rapid diagnostic malaria test (RDT) for all clinically suspected cases of malaria in pregnancy (case management), and treatment of uncomplicated *P. vivax* cases in pregnancy with chloroquine for three days, and uncomplicated *P. falciparum* cases with artesunate for three days in combination with single dose sulphadoxine-pyrimethamine on day 1. Quinine was the drug of choice for first trimester treatment of *P. falciparum*. All pregnant women with anaemia (haemoglobin [Hb] <11 g/dL) were given haematinic supplementation (usually Fefol tablets: a combination of 150 mg ferrous sulphate and 0.5 mg folic acid) in the antenatal clinics. Although at the time of the study weekly chloroquine prophylaxis was recommended, this was not implemented in the participating hospitals.

### Procedures

Surveys were conducted in the delivery units in a hospital in Jabalpur city between September and October 2006 and in hospitals in the towns of Katni (semi-rural) and Maihar (rural) between October 2006 and September 2007 to assess the burden of MiP. Information on demographic parameters, reproductive and malaria morbidity and prevention history during pregnancy, and delivery outcome were obtained. Newborns were weighed to the nearest 10 grams within two hours of delivery on a digital scale (Sansun, Sansui Electronics Pvt Ltd, India). Scales were calibrated weekly with a standard 5 kg weight. Gestational age was assessed within 24 hours of delivery using the Ballard score by staff trained by and under weekly supervision of the study paediatrician [28]. A maternal finger prick blood sample was collected during labour for malaria microscopy, pLDH-based rapid diagnostic test (RDT) (First Response Pf/Pv® from Premier Medical Corporation Ltd, Mumbai, India) and haemoglobin measurement (HemoCue® B-Hemoglobin, Angelholm, Sweden). All women with peripheral parasitaemia detected by microscopy or RDT were referred to the clinicians for appropriate treatment. Within three hours of delivery, placental incision and impression smears for microscopy and placental blood for PCR were taken. In addition, two 2 x 2 x 1cm biopsies for histopathology were taken from the maternal side of the placenta at opposite sites half-way between the umbilical cord insertion and the placental edge and fixed in 10% phosphate buffered formalin. Fixed tissues were trimmed to 3 mm sections, placed in cassettes and fresh buffered formalin and stored in an air-conditioned room with the fixative changed monthly to ensure pH stability. Specimens were embedded in paraffin wax within 3 months using standard techniques, cut into 5 μm sections, mounted on slides (two sections *per* placenta) and processed and stained with haematoxylin and eosin at the National Institute for Malaria Research (NIMR) field laboratory in Jabalpur, India.

Histology slides were examined locally under standard and polarized light microscopy to increase the sensitivity of haemozoin detection [29]. The reviewer (RA) was aware of the blood smear results, but unaware of the maternal characteristics, obstetric history and pregnancy outcome of the examined cases. Slides were examined under 40x, or 100x magnification for the presence of parasites and inflammatory cells and scored semi-quantitatively in any of the 50 fields examined. The scoring system used is presented in the Table 1. Based on histopathology, cases with parasites were classified as active infections, consisting of chronic infections (parasites plus haemozoin in monocytes and/or fibrin) and acute infection parasites and +/- pigmented monocytes with few spots or no pigmented fibrin [3,4]. Cases with pigment in fibrin in the absence of parasites were classified as past infections. Percentage parasitized erythrocytes was calculated against 500 maternal erythrocytes per high power (HPF, 100x magnification) and classified < 1% parasitaemia if 1–5 infected erythrocytes per HPF was observed (scanty), 1-10% in moderate infection and > 10% parasitaemia when >10 infected erythrocytes was observed in 2 or more HPF (Table 1). When there was less than 500 erythrocytes per HPF the formula used was number of parasitized erythrocytes multiplied by 100/number of maternal erythrocytes present per HPF.

**Table 1:** Scoring system used to evaluate presence and severity of placental pathologic changes and parasitaemia

	**Histopathologic severity**		
	**Mild**	**Moderate**	**Severe**
Perivillous or intervillous fibrin	Present in <20% IVS	Present in 20-50% IVS	Present in >50% IVS
Fibrinoid necrosis	Focal: localised to few fibrin areas		Diffuse: >50% fibrin affected
Calcification	Focal: localised to few areas of fields viewed		Diffuse: >50% affected in total fields viewed
Syncytial knots	Normal: <1/3 villi affected		>1/3 villi affected in total fields viewed
Pigment deposition	Focal presence in small amounts	Small spots or larger de- posits in many locations	Large amount present widely
Polymorphonuclear cells	<10 cells/HPF	10-25 cells/HPF	>25cells /HPF
Mononuclear cells	<10 cells/HPF	10-25 cells/HPF	>25 cells/HPF
Parasite presence*	**Scanty**: 1-5/in any HPF	5-10 in 2 or more HPF	>10 in any 2 or more HPF
Parasitaemia**	**1%**	1-10%	**>10%**

**Note**: Intervillous spaces and villous structures were examined for the presence of parasitized cells and inflammatory cells. The intervillous and perivillous fibrin, inflammatory cells, villous stroma, the syncytium and Hofbauer cells were examined for the presence of pigment deposition. Morphological changes were evaluated in syncytial knots, Hofbauer cells, foetal red blood cells, villous stroma and fibrinoid necrosis and calcification. Pigment was identified as brownish granular looking material that shows birefringence under polarized light. Calcifications were identified as dark bluish purple spots that did not show birefringence on polarized light.

*Plasmodium species can not be identified on histology slides, and is based on the standard microscopy and RDT results. **Percent parasitaemia is calculated against 500 erythrocytes per HPF.

*Abbreviations:IVS* intervillous space, *HPF* High power field (100x), (total fields viewed = 50), *RDT* rapid diagnostic tests.

PCR of placental blood was performed for species confirmation in all the 52 histology positive cases, and for an additional 63 negative cases matched for site and gravidity, selected for quality assurance for a malaria diagnosis sub-study. Placental red blood cell pellets produced at the time of placental sampling from 250-500 μL blood and stored at -20°C were analysed. Nested PCR was used for genus and species specific analysis of *P. falciparum* and *P. vivax;* PCR analysis was carried out at the NIMR field station laboratory in Jabalpur [30]. The primary reaction was conducted on a volume of 25 μL consisting of 5 μL DNA. Species amplification was performed on aliquot of 2 μL in 1:10 dilution of first reaction mixture.

### Definitions

Subpatent infections were defined as active infections detected by histopathology which were negative by any standard peripheral and placental microscopy and peripheral RDT. A low birth weight was defined as a birth weight <2,500 grams among singleton deliveries. Prematurity was defined as a new born delivered before the gestational age of 37 weeks among singletons as defined by the Ballard score. Fever at the time of delivery was defined as documented fever with an axillary temperature of ≥37.5°C, or a history of fever in the past 24 hours at the time of delivery.

### Sample size

Because of costs and labour limitations, not all placental histology samples could be processed. A sub-sample of 506 from 2,282 participants was selected for placental histology, including all 50 malaria positive cases (2.2% of total sample) at delivery determined by microscopy or RDT on either peripheral, or placental smear (incision or impression smear). The remaining 456 samples were randomly selected from the remaining 2,232 smear/RDT negative women stratified by site. For every woman with a patent infection (smear or RDT positive) at least one additional infection was assumed to be detected by histology (active or past infection). A sample size of 456 smear/RDT negative cases allowed us to detect a prevalence of sub-patent infections of ≤3% with an absolute precision of ±3% at a 95% confidence level.

### Statistical analysis

Data was analysed using SAS® 9.1 (Statistical Application Software, SAS Institute Inc. Cary NC). Placental changes corresponding to infection categories were evaluated using the *χ*^*2*^ test. To assess the impact of infection on birth outcomes, univariate analyses were performed using Student’s *t* test or analysis of variance (ANOVA) for continuous endpoints. Multivariate analyses were performed using General Linear Models (GLM) with birth weight, gestational age and haemoglobin as the continuous dependent variables and histology graded infection status (acute, chronic, past or no infection) as the independent predictor of interest. Additional models assessed the association between these endpoints and the individual or combination of histological parameters (parasites, syncytial knots, fibrinoid necrosis, calcification, intervillous space polymorphonuclear cells, intervillous space monocytes, intervillous space fibrin). The socio-demographic parameters; socioeconomic status, education, residence and gravidity were included in all initial models as co-variates. A significance level of *P* = 0.05 was used to retain independent predictors in the model and 95% confidence intervals were calculated using Tukey’s multiple comparison test.

### Ethical approval

The study was approved by the Research Ethical Committees of the National Institute of Malaria Research Delhi, Indian Council of Medical Research in India, CDC in U.S.A, and the Liverpool School of Tropical Medicine (LSTM) in the UK. All the study participants provided written informed consent.

## Results

The mean age of women was 23.8 (3.4) years and there were 42.7% primigravidae (Table 2). The proportion of women reporting use of bed net the previous night was 17.8%. A documented fever (≥37.50C) was present in 6.9% women and 16.2% reported fever previous week, whereas only 1.9% used anti-malarial for fever. Low birth weight was 37% and 20.8% was preterm births. In total there were 22 (4.4%) stillbirths out of which 4 cases were in women with placental malaria.

**Table 2:** Participant characteristics

**Parameters**	**N = 506**
Age in years, mean (SD)	23.8 (3.4)
**Age categories**	
<20 yr, n (%)	10 (1.9)
20-29 yr, n (%)	454 (89.9)
≥30 yr, n (%)	41 (8.1)
**Residence**	
Urban, n (%)	148 (29.2)
Rural, n (%)	359 (70.8)
**Education**	
No schooling, n (%)	161 (31.8)
Primary, n (%)	136 (26.8)
Secondary, n (%)	173 (34.2)
Higher, n (%)	36 (7.1)
**SES**^**1 **^**rank (quintiles) n (%)**	
1 – Poorest	117 (23.1)
2	107 (21.2)
3	112 (22.1)
4	85 (16.8)
5	85 (16.8)
**Gravidity**	
Primigravidae, n (%)	216 (42.7)
Secundigravidae, n (%)	162 (32.0)
Gravidae-3, n (%)	73 (14.4)
Gravidae-4, n (%)	34 (6.7)
Multigravidae (≥5)^2^ n (%)	21 (4.2)
Used bednet previous night, n (%)	89 (17.8)
ITN (any), n (%)	1 (0.2)
Used any drug to prevent malaria, n (%)	4 (0.8)
Reported using haematinics, n (%)	427 (84.7)
**Morbidity and Outcomes**	
Reported fever previous week, n (%)	82 (16.2)
Used antimalarial for fever, n (%)	10 (1.9)
Maternal fever at delivery^3^, n (%)	35 (6.9)
Birth weight^±^ (g), mean (SD)	2621 (450)
Birth weight <2500 g, n (%)	176 (37.1)
Baby’s gestational age in weeks^4^, mean, (SD)	37.2 (1.3)
Preterm babies <37 weeks), n (%)	95 (20.0)
Stillbirths^≠^, n (%)	22 (4.4)
Stillbirths in uninfected women, n (%)	18 (3.6)
Maternal Hb (g/dL), mean (SD)	10.5 (2.2)

^1^SES = socioeconomic status (based on underlying survey population); ^2^= pregnancy number.

^3^= Fever = axillary temperature ≥ 37.5°C; ^4^= assessed by Ballard score; ^±^Birth weight and gestational age data was available for 474 babies out of 482 singleton live births; data for 2 babies are missing out of the 506 samples; ^≠^Stillbirth denominator = 504.

### Malaria infection based on histopathology

Placental malaria was identified in 52 of the 506 cases (Table 3). Of these, 38 (73.1%) were active infections (20 acute infections plus 18 chronic infections) and 14 (26.9%) were past infections. Out of the histology positive samples, 38 were positive by PCR (32 active infections and six past infections). There were 11 active infection (2.4%) and 11 (2.4%) past infections detected by placental histology that were negative by the other test techniques.

**Table 3:** Histopathology based placental malaria infections compared to standard microscopy and other tests

	**Histopathology**					
		Infected*	**Acute**	**Chronic**	**Past**	**Non-infected**
**Tests**	**n/N**	**n = 38**	**n = 20**	**n = 18**	**n = 14**	**n = 454**
**Any species**						
Any smear/RDT+, n (%)	47/505^a^	27/38 (71.1)	20/20 (100)	7/18 (38.9)	3/14 (21.4)	17/453 (3.7)
Peripheral microscopy+, n (%)	39/504^b^	25/38 (65.8)	20/20 (100)	5/18 (27.8)	2/14 (14.3)	12/453 (2.6)
Placenta impression smear+, n (%)	22/505^c^	17/38 (44.7)	14/20 (70.0)	3/18 (16.7)	1/14 (7.1)	4/453 (0.9)
Placental incision smear+, n (%)	32/504^d^	23/37 (62.1)	18/20 (90.0)	5/17 (29.4)	2/14 (14.3)	7/453 (1.5)
RDT+, n (%)	29/506	20/38 (52.6)	17/20 (85.0)	3/18 (16.7)	1/14 (7.1)	8/454 (1.7)
No infection smear/RDT, n (%)	458/505	11/38 (28.9)	0/20 (0)	11/18 (61.1)	11/14 (78.6)	436/453 (96.2)
PCR+, n (%)	62/110^e^	32/38 (84.2)	19/20 (95.0)	13/18 (72.2)	6/14 (54.5)	24/62 (38.7)
***P. falciparum***						
Any smear/RDT+, n (%)	24/505^‡^	21/38 (55.3)	14/20 (70.0)	7/18 (38.9)	0	3/453 (0.6)
Peripheral microscopy, n (%)	28/505	25/38 (65.8)	20/20 (100)	5/18 (17.9)	0	3/453 (0.7)
Placental impression smear, n (%)	19/505	17/38 (44.7)	14/20 (70.0)	3/18 (15.8)	0	2/453 (0.4)
Placental incision smear, n (%)	26/504	23/37 (60.5)	18/20 (90.0)	5/18 (19.2)	0	3/453 (0.7)
RDT+, n (%)	12/506	11/38 (28.9)	9/20 (45.0)	2/18 (11.1)	0	1/454 (0.2)
PCR+, n (%)	45/110^f^	30/38 (78.9)	18/20 (90.0)	12/18 (66.6)	3/11 (21.4)	12/62 (19.4
***P. vivax***						
Any smear/RDT+, n (%)	14/505^‡^	0	0	0	3/14 (21.4)	11/453 (2.4)
Peripheral microscopy, n (%)	11/505	0	0	0	2/14 (14.3)	9/453 (2.0)
Placental impression smear, n (%)	3/504	0	0	0	1/14 (7.1)	2/453 (0.4)
Placental incision smear, n (%)	6/505	0	0	0	2/14 (14.3)	4/453 (0.9)
RDT+, n (%)	8/506		2/20 (10.0)	1/18 (5.5)	1/14 (7.1)	4/454 (0.8)
PCR+, n (%)	10/110^f^	0	0	0	3/14 (21.4)	7/62 (11.3)
**Mixed infection (Pf + Pv)**						
RDT mixed +, n (%)	9/506	6/38 (15.8)	6/20 (30.0)	0	0	3/454 (0.7)
PCR mixed +, n (%)	7/110	2/38 (5.3)	1/20 (5.0)	1/18 (5.6)	0	5/62 (8.1)

^a^Out of 50 any smear/RDT positives, 3 samples were missing and were not included in the analysis; *acute + chronic infections ^‡^excludes mixed RDT infections ^b^Among 39 positive peripheral smears, 11 were incision negative, and 20 were impression negative. ^c^Among 22 positive impression smears, 3 were peripheral smear negative; ^d^Among 32 positive incision smears, 4 were peripheral smear negative; ^e^PCR was performed for species confirmation on a sub-set of 115 samples corresponding to histology positive samples and a similar number of negative samples chosen for quality assurance matched by gravidity and study sites; ^f^Includes mixed species; there were no mixed species infection detected on smear microscopy. Abbreviations: Plac. = placental.

Among the 38 active infections identified by histology, 25 (65.8%, 95% CI 48.5-79.8) were positive by peripheral smear microscopy. These included all 20 acute infections and five of the 18 chronic infections (Table 3). All 25 infections corresponded to *P. falciparum* and none was due to *P. vivax*. None of the 14 cases with histological evidence of a past infection had detectable *P. falciparum* malaria by peripheral microscopy, but two were peripheral microscopy positive for *P. vivax*.

Incision smears identified 60.5% (95% CI 44.7-77.1) of histological *P. falciparum* placental infections (Table 3). The positive predictive value of an incision smear to detect *P. falciparum* placental infection was 88.5% (95% CI 68.7-96.9) and the negative predictive value was 96.9% (94.8-98.2). Impression smears correctly identified placental infection in 44.7% (28.9-61.5) of cases, with a positive predictive value of 89.4% (65.4-98.1), and a negative predictive value of 95.4% (93.0- 97.1). Combined positivity of either incision or impression smear identified 70% of histopathologically active infections.

The distribution of parasitized erythrocytes in the intervillous space was scanty: 60% showed <1% parasitaemia and 32.5% cases had parasitaemia between 1-10%. Individual erythrocytes were rarely seen adhering to the syncytial layer. No parasites were observed in foetal erythrocytes or within the villous structures. Among the active infections, monocyte presence was mostly mild (63%) or moderate (23.6%) with only 1 case of a severe intervillous infiltrate (2.6%) (Table 4). The geometric mean parasite density of *P. falciparum* was 568 parasites/μL and *P. vivax* was 149 parasites/μL in maternal blood samples corresponding to the placental histology samples (not shown in the table).

**Table 4:** Placental histology features by category of histopathological infection (independent of Plasmodium species)

**Placental features**	**No**^**†**^	**Past**^**††**^	**Past vs**	**Active**^**♣**^	**Active *vs *** **No inf.**	**Acute Inf.**	**Acute *vs *** **No inf.**	**Chronic Inf.**	**Chronic *vs *** **No inf.**
	**Inf.**	**Inf.**	**No inf.**	**Inf.**					
	**n = 454**	**n = 14**	***p*-value**	**n = 38**	***p *** **value**	**n = 20**	***p *** **value**	**n = 18**	***p *** **value**
**Syncytial Knots**									
Normal, n (%)	414 (91.2)	8 (57.1)	<0.001	18 (47.4)	<0.0001	12 (60.0)	0.0003	6 (33.3)	<0.0001
Increased (>1/3), n(%)	40 (8.8)	6 (42.9)		20 (52.6)		8 (40.0)		12 (66.7)	
**Fibrinoid necrosis**									
Absent, n (%)	393 (86.5)	7 (50.0)		27 (71.1)		20 (100.0)		7 (38.9)	
Focal, n (%)	58 (12.8)	6 (42.9)	0.0001	9 (23.6)	0.003	0	0.15	9 (50.0)	<0.0001
Diffuse, n (%)	3 (0.7)	1 (7.1)		2 (5.3)		0		2 (11.1)	
**Calcification**									
Absent, n (%)	416 (91.6)	9 (64.3)		29 (76.3)		20 (100.0)		9 (50.0)	
Focal, n (%)	31 (6.8)	4 (28.6)	0.002	7 (18.4)	0.008	0	0.30	7 (38.9)	<0.0001
Diffuse, n (%)	7 (1.6)	1 (7.1)		2 (5.3)		0		2 (11.1)	
IVS* PMN cell									
Absent, n (%)	340 (74.9)	13 (92.9)		26 (70.3)		14 (70.0)		12 (70.6)	
Mild, n (%)	80 (17.6)	1 (7.1)	0.47	9 (24.3)	0.75	4 (20.0)	0.93	5 (29.4)	0.45
Moderate, n (%)	33 (7.3)	0		2 (5.4)		2 (10.0)		0	
Severe, n (%)	1 (0.2)	0		0		0		0	
IVS* monocyte									
Absent, n (%)	358 (78.8)	11 (78.6)		3 (7.9)		1 (5.0)		2 (11.1)	
Mild, n (%)	74 (16.3)	3 (21.4)	0.63	24 (63.2)	<0.0001	12 (60.0)	<0.0001	12 (66.7)	<0.0001
Moderate, n (%)	22 (4.9)	0		10 (26.3)		6 (30.0)		4 (22.2)	
Severe, n (%)	0	0		1 (2.6)		1 (5.0)		0	
IVS* fibrin									
Absent, n (%)	134 (29.5)	0		4 (10.5)		4 (20.0)		0	
Mild, n (%)	268 (59.0)	5 (35.7)	<0.001	25 (65.8)	0.0002	15 (75.0)	<0.47	10 (55.6)	<0.0001
Moderate, n (%)	52 (11.5)	9 (64.3)		8 (21.1)		1 (5.0)		7 (38.9)	
Severe, n (%)	0	0		1 (2.6)		0		1 (5.5)	

All are column %. Active infection group includes 1 missing result for IVS PMN cell score. ^**†**^histology classification. No-infection = parasite and pigment absent, Acute infection = parasite present, ±pigmented monocytes, ± few spots pigment in fibrin; Chronic infection = parasite present, ±pigmented monocytes, +pigment in fibrin; Active infection = acute + chronic infection; Past infection = no parasites, only pigment present; ^**††**^includes 2 cases corresponding to *P. vivax* infection; ^♣^pooled histology classified acute & chronic infection. Abbreviations: ^*^IVS = intervillous space; PMN = polymorphonuclear.

### Histopathological changes associated with malaria

Active and past infections were associated with an increased frequency of syncytial knotting, focal fibrinoid necrosis, focal calcification and fibrin deposits in the intervillous space (Table 4). Signs of severe infection or inflammation were not observed. The placental histological features corresponding to PCR confirmed peripheral blood mono-infections of *P. falciparum* (n = 28) or *P. vivax* (n = 11) are presented in Table 5. *Plasmodium falciparum* mono-infections were more likely to be associated with mild to moderate infiltration of intervillous monocytes and fibrin deposits than *P. vivax* mono-infection, whereas *P. vivax* infections were associated with increased syncytial knots and moderate polymorphonuclear cell infiltration.

**Table 5:** **Placental histology features in peripherally patent *P. falciparum *** **and *P. vivax *** **cases**

**Placental feature**	**No**^**† **^**infection**	**Pf**	**Pv**	**Pf *vs *** **no infection**	**Pv *vs *** **no Infection**	**Pf *vs *** **Pv**
	**n = 441**	**n =28**	**n = 11**	***P *** **value**	***P *** **value**	***P *** **value**
**Syncytial Knots**						
Normal, n (%)	406 (92.1)	16 (57.1)	4 (36.4)	<0.0001	<0.0001	0.03
Increased (>1/3), n (%)	35 (7.9)	12 (42.9)	7 (63.6)			
**Fibrinoid necrosis**						
Absent, n (%)	380 (86.2)	24 (85.7)	9 (81.8)			
Focal, n (%)	58 (13.2)	3 (10.7)	2 (18.2)	0.25	0.85	0.68
Diffuse, n (%)	3 (0.6)	1 (3.6)	0			
**Intravillous calcification**						
Absent, n (%)	403 (91.4)	25 (89.3)	11 (100.0)			
Focal, n (%)	31 (7.0)	2 (7.1)	0	0.73	0.59	0.52
Diffuse, n (%)	7 (1.6)	1 (3.6)	0			
**Intervillous fibrin**						
Absent, n (%)	125 (28.3)	6 (21.4)	7 (63.6)			
Mild, n (%)	265 (60.1)	20 (71.4)	4 (36.4)	0.48	0.03	0.03
Moderate, n (%)	51 (11.6)	2 (7.2)	0			
Severe, n (%)	0	0	0			
**IVS polymorphonuclear cells**						
Absent, n (%)	335 (75.9)	19 (70.4)	3 (27.3)			
Mild, n (%)	77 (17.5)	6 (22.2)	3 (27.3)	0.91	<0.0001	0.01
Moderate, n (%)	28 (6.4)	2 (7.4)	5 (45.4)			
Severe, n (%)	1 (0.2)	0	0			
**Intervillous monocytes**						
Absent, n (%)	351 (79.6)	4 (14.3)	5 (45.5)			
Mild, n (%)	69 (15.7)	13 (46.4)	5 (45.5)	<0.0001	0.02	0.12
Moderate, n (%)	21 (4.7)	10 (35.7)	1 (9.0)			
Severe, n (%)	0	1 (3.6)	0			

All are column %; ^**†**^negative for parasites by smear microscopy & histology. Abbreviations: *Pf = P. falciparum*, *Pv = P. vivax;* IVS = intervillous space.

### Impact of histological changes (*Plasmodium falciparum*) on maternal morbidity and delivery outcome

Among the 506 cases examined for histopathology, there were 22 stillbirths. Four stillbirths occurred in women with an active infection based on histology (4/38 = 10.5%), which was significantly higher than in uninfected women (RR 3.8, 95% CI 1.2-12.1). Three of the four had parasitaemia <1% (where placental microscopy identified 1–5 parasites per high power field), and one infection was classified as severe. Chronic infections were not associated with an increase in stillbirth (RR 1.3, 95% CI 0.17-9.1).

Overall, 21.5% of women had fever (temperature ≥ 37.5°C) at the time of delivery or history of fever in the previous week. Fever on the day of delivery was not associated with active placental infections (RR 0.8, 95% CI 0.27-2.6) whereas relative risk of an active placental infection was 2.5 (95% CI 1.5-4.0) in women with history of fever in the past week. Fever was not associated with stillbirth however; fever occurred in 27.3% [6/22] of women with a stillbirth, and 20.7% [98/473] of women with a live birth, RR 1.3, 95% CI 0.65-2.6).

There were 482 singleton live births. The presence of parasites or pigmented monocytes was associated with decreased birth weight, gestational age at birth and maternal haemoglobin levels in univariate analysis (Table 6). In addition, increased syncytial knotting or focal calcification was associated with a shorter duration of pregnancy. Multivariate models showed that only the acute *P. falciparum* infections were associated with reductions in birth weight (400 g, 95% CI 113–687) or mean gestational age (0.8 weeks, 95% CI 0.04-1.6) or lower haemoglobin levels (1.4 g/dL, 95% CI 0.5-2.4) relative to no-infection (Table 7). Further analysis showed an interaction between the effects of pigmented monocytes and parasites. The greatest effect on birth weight, gestational age and haemoglobin were observed among women who had both parasites and pigmented monocytes, whereas the effect of the presence of parasites only (n = 17) or of pigmented monocytes only (n = 14) was smaller and not statistically significant (Table 7). Further stratification showed that the reduction of birth weight was greatest in women with moderate-to-severe infiltrates of pigmented monocytes, as opposed to mild levels of infiltration (Table 7).

**Table 6:** **Univariate analysis of placental *Plasmodium falciparum *** **histological determinants of birth weight, gestational age and maternal haemoglobin**

		**Birth weight (BW)**			**Gestational age (GA)**			**Maternal haemoglobin (Hb)**			
**Factors**	**N**	**Mean BW (SD)**	**Mean difference (95% CI)**	***p***	**Mean GA (SD)**	**Mean difference (95% CI)**	***p***	**N**	**Mean Hb (SD)**	**Mean difference (95% CI)**	***p***
**Placental parasites**											
Present	33	2419 (535)	-217 (-375, -58)	0.01	36.7 (1.2)	-0.5 (-1.03, -0.11)	0.01	38	9.6 (2.4)	-0.9 (-1.63, -0.17)	0.02
Absent	441	2636 (440)	Reference		37.3 (1.3)	Reference		464	10.5 (2.1)	Reference	
**Syncytial knots**											
Increased	59	2546 (501)	-85 (-208, 37)	0.2	36.8 (1.6)	-0.5 (-0.81, -0.1)	0.01	65	10.3 (2.6)	-0.2 (-0.79, 0.35)	0.4
Normal	415	2631 (442)	Reference		37.3 (1.2)	Reference		437	10.5 (2.1)	Reference	
**Fibrinoid necrosis**											
Present	75	2620 (476)	-1 (-112, 110)	0.9	37.0 (1.4)	-0.3 (-0.58,0.05)	0.1	78	10.6 (2.2)	0.2 (-0.32, 0.76)	0.4
Absent	399	2621 (446)	Reference		37.3 (1.3)	Reference		424	10.4 (2.2)	Reference	
**Intervillous fibrin**											
Moderate-severe	68	2616 (432)	-18 (-178, 141)		37.1 (1.5)	-0.1 (-0.5, 0.4)		68	10.2 (1.8)	-0.6 (-1.34, 0.18)	0.1
Mild	281	2616 (470)	-18 (-133, 95)	0.8	37.2 (1.3)	0.0 (-0.3, 0.3)	0.8	295	10.4 (2.3)	-0.4 (-0.90, 0.16)	
Absent	125	2634 (417)	Reference		37.2 (1.2)	Reference		139	10.8 (2.1)	Reference	
**Calcification**											
Focal	51	2519 (426)	-114 (-245, 16)	0.1	36.8 (1.3)	-0.5 (- 0.87, -0.11)	0.01	51	10.2 (2.8)	-0.3 (-1.03, 0.24)	0.2
Absent	423	2633 (452)	Reference		37.3 (1.3)	Reference		451	10.5 (2.1)	Reference	
**Pigmented fibrin**											
Present	36	2527 (452)	-101 (-254, 52)	0.2	37.0 (1.2)	-0.3 (-0.72, 0.16)	0.2	40	10.2 (2.1)	-0.3 (-0.98, 0.44)	0.4
Absent	438	2628 (450)	Reference		37.3 (1.3)	Reference		462	10.5 (2.2)	Reference	
**Pigmented monocytes**											
Present	16	2257 (516)	-376 (-598, -153)	0.001	36.5 (1.2)	-0.7 (-1.37, -0.07)	0.02	19	9.1 (2.7)	-1.4 (-2.4, -0.4)	0.01
Absent	458	2633 (443)	Reference		37.3 (1.3)	Reference		483	10.5 (2.1)	Reference	
^*^IVS Polymorponuclear cells											
Moderate-severe	33	2594 (422)	-22 (-215, 172)		37.5 (1.1)	0.3 (-0.22, -0.89)		36	10.8 (2.5)	0.3 (-0.54, 1.26)	0.5
Mild	84	2647 (436)	31 (-98, 159)	0.8	37.3 (1.3)	0.1 (-0.21,0.53)	0.2	90	10.4 (2.3)	-0.1 (-0.70, 0.51)	
Absent	356	2616 (458)	Reference		37.2 (1.3)	Reference		376	10.5 (2.1)	Reference	
^*^IVS Monocytes											
Moderate-severe	30	2634 (515)	-1 (-202, 200)		37.4 (1.6)	0.2 (-0.42,0.74)		33	9.7 (2.7)	-0.8 (-1.79, 0.08)	
Mild	95	2563 (454)	-72 (-195, 50)	0.4	37.1 (1.2)	-0.1 (-0.46, 0.25)	0.6	101	10.5 (2.4)	-0 .1 (-0.65, 0.51)	0.1
Absent	349	2635 (444)	Reference		37.2 (1.3)	Reference		368	10.6 (2.1)	Reference	

Abbreviations: SD = standard deviation; CI = confidence interval; ^*^IVS = intervillous space; BW = Birth weight; GA = gestational age

**Note**: BW and GA were assessed for singleton live-birth babies only. Among 506 cases assessed, BW and GA were not available for 23 stillbirths, 1 twin delivery, and 8 newborn babies who needed immediate medical attention at birth. Maternal haemoglobin was missing in 4 of the 506 cases.

**Table 7:** Association of placental falciparum histologically-confirmed infection and birth weight, gestational age and maternal haemoglobin – multivariate analysis

		**Adjusted birth weight**			**Adjusted gestational age**			**Adjusted maternal haemoglobin**			
**Factors**	**N**	**Mean (SD)**	**Mean difference (95% CI)**	***P***	**Mean GA (SD)**	**Mean difference (95% CI)**	***P***	**N**	**Mean Hb (SD)**	**Mean difference (95% CI)**	***P***
**1. Infection based on histopathology**[3]											
Acute	16	2232 (541)	-400 (-687, -113)	0.001	36.5 (1.3)	-0.8 (-1.6,-0.04)	0.06	20	8.8 (2.4)	-1.4 (-2.4,-0.5)	0.02
Chronic	17	2594 (481)	-38 (-316, 240)		37.0 (1.2)	-0.3 (-1.2, 0.45)		18	10.6 (2.2)	0.1(-0.8, 1.1)	
Past	12	2820 (518)	188 (-141, 517)		37.3 (1.1)	0.0 (-0.9, 1.0)		11	10.4 (2.1)	-0.1(-1.5,1.0)	
No infection	419	2632 (442)	Reference		37.3 (1.3)	Reference		442	10.5 (2.1)	Reference	
**2. Placental infection based on**											
Para + p monoc	16	2274 (516)	-356 (-580, -132)	0.01	36.6 (1.2)	-0.6 (-1.3, 0.02)	0.04	19	9.3 (2.7)	-1.1 (-2.1, -0.1)	0.016
Parasites only	17	2588 (523)	-42 (-257, 173)		36.7 (1.3)	-0.5 (-1.2, 0.07)		19	10.2 (2.1)	-0.2 (-1.2, 0.8)	
No infection	431	2630 (444)	Reference		37.3 (1.3)	Reference		453	10.4 (2.2)	Reference	
**3. Placental infection based on**											
Para + ms p monoc	3	2031 (582)	-600 (-1108,-92)	0.01	36.2 (1.0)	-1.1 (-2.6, 0.3)	0.05	4	9.1 (1.5)	-1.4 (-3.4, 0.8)	0.041
Para + mi p monoc	13	2329 (507)	-302 (-547, -56)		36.7 (1.2)	-0.6 (-1.2, 0.2)		15	9.2 (2.9)	-1.3 (-2.2, 0.1)	
Parasites only	17	2588 (523)	- 43 (-258,173)		36.7 (1.4)	-0.6 (-1.2, 0.1)		19	10.2 (2.2)	-0.3 (-1.2, 0.7)	
No infection	431	2631 (444)	Reference		37.3 (1.3)	Reference		453	10.5 (2.2)	Reference	
4. Sub-patent infections*											
Para + p monoc	3	2446 (490)	-177 (-616, 263)	0.4	37.0 (0.8)	-0.3 (-1.6, 0.9)	0.8	4	10.4 (4.1)	-0.1 (-2.1, 2.1)	0.9
Parasites only	8	2778 (430)	155 (-151, 461)		37.2 (1.4)	-0.1 (-1.0, 0.8)		8	10.7 (1.5)	-0.2 (-1.1, 1.6)	
No infection	431	2623 (444)	Reference		37.3 (1.3)	Reference		453	10.5 (2.1)	Reference	

SD = standard deviation; CI = confidence interval; *P* = p-value; para = parasite; p = pigmented; mi = mild; ms = moderate to severe; monoc = monocytes. All models adjusted for socioeconomic status, gravidity, education, residence. *Model 4 = histology positive, among those negative by any of the standard microscopy/RDT methods.

Histopathology did not identify any active *P. vivax* infections. The presence of peripheral *P. vivax* infection (11 cases) was not associated with significant reductions in birth weight (2561 g [SD 200] [*P. vivax*, n = 11] vs. 2639 gr [SD 445] [no *P. falciparum* or *P. vivax* malaria, N = 445], mean difference: -78 g, 95% CI -225.2 to 69.1), a reduced gestational age (-0.3 weeks, 95% CI -1.1 to 0.5) or maternal haemoglobin (-0.4 g/dL, 95% CI -1.6 to 0.9).

### Sub-patent placental infections (any smear or RDT negative, histology positive)

In the 12 women who were slide smear and peripheral RDT negative but who had parasites detected by histology (sub-patent infection), the mean birth weight, gestational age and maternal haemoglobin levels were lower if both parasites and pigmented monocytes were detected, but numbers were small and the difference was not statistically significant (Table 7).

## Discussion

Few studies have described placental malaria associated histopathology outside sub-Saharan Africa. Histological changes associated with *P. falciparum* and *P. vivax* infections and the corresponding impact on adverse birth outcome and maternal anaemia were assessed in an area of low malaria transmission in central India. This was a pregnant population at low risk of malaria and despite the low parasitaemia seen by the low maternal blood geometric mean parasite density and placental parasite percentage, the impact of infection was considerable and independent of the presence of documented of fever.

Based on placental incision microscopy, the overall prevalence of malaria at delivery was 1.0%, and comparable to recent studies in other regions in India [31,32]. As expected, placental histology was more sensitive to detect *P. falciparum* malaria than placental or peripheral smear microscopy alone. Approximately one in 40 women (2.4%) that were negative by conventional microscopy (either placental or peripheral) or pLDH-based RDT were found to have active placental *P. falciparum* infections (acute or chronic) by histology. When this estimate of ‘subpatent’ infections was extrapolated to the overall survey sample of 2282 women, the prevalence of *P. falciparum* placental malaria increased from 2.2% (50/2282) to 4.5%, doubling the estimate. Placental incision microscopy alone missed about two out of every three infections which could be explained by the largely low parasitaemia. Women with subpatent infections had lower maternal haemoglobin, mean birth weight and gestational age, although the difference did not reach statistical significance, likely because of the small number of women with submicroscopic infections.

Most (89.3%, 25/28) of the women with patent maternal blood *P. falciparum* infections detected by conventional microscopy also had evidence of an active placental histopathology infection. In contrast, no placental parasites could be detected by histology among the 11 women with patent peripheral *P. vivax* infections and none of the women who were smear negative for peripheral *P. vivax* had evidence of infection with histopathology. This highlights the importance of using sensitive methods such as placental histology or PCR to detect placental *P. falciparum* infection as conventional peripheral and placental microscopy smear results alone may markedly underestimate the true burden of *P. falciparum* malaria in this region.

Histopathology is a useful method to grade the chronology of placental *P. falciparum* infections [3,4]. Compared to observations in areas of high transmission, a relatively large proportion (38%) of histopathological infections with *P. falciparum* were acute rather than chronic or past infections. This might be explained by the low malaria exposure and low host immunity in this population triggering an inflammatory response and early delivery when the placenta becomes infected, although the adverse impact of infection was independent of the occurrence of fever in this study. The low exposure is also reflected by the low parasitaemia within the placenta and the corresponding mild inflammatory response. This contrasts with the dense parasitaemia and the occasionally massive chronic intervillositis and monocyte infiltration reported from highly endemic areas in sub-Saharan Africa [9], but is similar to observations from the Thai-Burmese border [10] and Colombia [16-18]. Similar to previous reports, most parasites were found in the intervillous space without adhering to the syncytial layer and none were observed within the villous structure [4,10,33].

Chronic *P. falciparum* infection was associated with an increase in syncytial knots, focal fibrinoid necrosis and calcification. These changes were also evident in past infections, suggesting they persist after parasites are cleared [4]. Alternatively, there might have been insufficient time for their resolution since time between infection and delivery might have been short, similar to observations on the Thai-Burmese border [10].

Placental *P. falciparum* malaria was associated with maternal anaemia. Acute infection was associated with lower levels of mean haemoglobin (1.4 g/dL), comparable to observations in a study from Malawi [7]. Chronic infections were not associated with lower haemoglobin level. The proportion of low birth weight in this population is high and is consistent with previous reports from India [34,35]. Adding to it, the impact of placental *P. falciparum* infection on birth weight was considerable, despite the scarcity of parasites within the placenta and the mild degree of placental inflammation. Of all the histological measures, only the presence of parasites and pigmented monocytic infiltration were consistently associated with reduced birth weight and shorter gestation in multivariate analysis. Further analysis showed that the effect on birth weight and gestation was particularly evident among the active infections with pigmented monocyte infiltration, a reduction in birth weight of 356 grams compared to 42 grams in women with presence of parasites alone, especially if this monocyte infiltration was moderate to severe. While mononuclear cells as a source of inflammatory cytokines have been suggested to have a role in poor foetal growth [11,36][37-39] the infection level of 1–5 parasites per 100x magnification field approximating to <1% parasitaemia in 60% of cases in this study also suggests that mechanisms other than the presence of placental parasitaemia *per se* may play a role.

The observed shortening of the duration of pregnancy with active infections was an important contributor to the reduction in birth weight. It is possible that with the increased risk of acute infections in women with history of fever, the inflammatory response to infections might induce early labour. Thus infections may not last to lead to chronicity. Alternatively clinical symptoms may lead women to seek treatment and prevent chronicity. The average duration of pregnancy was 4 days shorter among women with an active infection and mild monocyte infiltration, and 8 days shorter in women with moderate to severe infiltration. Although this may appear a relatively small difference, the average increase in birth weight between 37 to 40 weeks gestation is estimated at 169 grams per week [40]. Additionally, chronic or past infections were not associated with low birth weight or shorter gestation, which supports the notion that malaria associated low birth weight results, at least in part, from a shortening of gestation triggered by acute infections in women with low protective immunity [4,7,9,41].

There was no evidence of *P. vivax* sequestration in the placenta. Pigment in the fibrin was observed in two peripherally patent *P. vivax* cases, which is consistent with reports from Thailand and Peru [10,24], although previous infections with *P. falciparum* cannot be excluded. Recent studies on immune aspect of placental malaria in Colombia indicate a possible pathogenic role of *P. vivax*[16,18] A noteworthy observation in *Plasmodium vivax* cases was the significantly higher presence of polymorphonuclear leukocytes and syncytial knots compared to *P. falciparum* infected placentas, suggesting a possible placental inflammatory response that might be different from that observed with *P. falciparum*. In addition, decreased fibrin deposition was seen compared to *P. falciparum* or uninfected placentas. There was no association, however, between polymorphonuclear cells or increased syncytial knots and birth weight or preterm births, although numbers may have been too small.

The study was limited by its cross-sectional design, which did not allow assessing the impact of earlier pregnancy infections via peripheral microscopy. If such infections resolve and are no longer evident as ‘past’ infections but still impact on maternal anaemia and birth outcome, the burden of malaria may have been underestimated. The number of women with *P. vivax* mono-infections was too low to draw firm conclusions. Although a single reader examined the sections and she was not blinded to the peripheral, placental microscopy and RDT results, a random sample of positive slides and those corresponding to patent *P. vivax* cases were reviewed blinded by a senior placental histopathologist in the US and confirmed the accuracy of the results of the first reading. PCR conducted in a subset of samples, provided further confirmation of species and the accuracy of the histological examination. The Ballard score that was used has not been validated in India. It was beyond the scope of this study to assess conditions other than malaria that may affect birth weight, haemoglobin level and prematurity (e.g. iron deficiency, other infections).

## Conclusions

Severe placental inflammation associated with *P. falciparum* infection proved rare in this population. Despite this, a substantial impact of *P. falciparum* placental infections on birth weight was observed, which in part could be explained by shortening in the average duration of pregnancy. There was no evidence of *P. vivax* sequestration in the placenta and any histological changes observed were mild, however the number of *P. vivax* infections were small. These findings with *P. falciparum* infections highlight the need for early (including subpatent) case-detection and the challenge of malaria control during pregnancy in areas of low malaria transmission to reduce the detrimental impact of malaria during pregnancy in this region.

## Competing interests

The authors declare that they have no competing interests.

## Authors’ contributions

RA, DJT, FTK, VU, NS and MD conceived and designed the study. RA led the overall surveys, conducted the histopathology component, data analysis and interpretation under supervision of DJT and FTK. PB and PS conducted the PCR component, RA wrote the first draft of the manuscript with input from DJT and FTK. All authors contributed to the final manuscript. All authors have contributed to, seen, and approved the final, submitted version of the manuscript.
